# Severe Hypoglycemia Caused by Recurrent Sarcomatoid Carcinoma in the Pelvic Cavity: A Case Report

**DOI:** 10.1097/MD.0000000000001577

**Published:** 2015-10-23

**Authors:** Chao Fang, Chuan Wen Fan, Yong Yang Yu, Cun Wang, Lie Yang, Yuan Li, Xian Ming Mo, Zong Guang Zhou

**Affiliations:** From the Department of Gastrointestinal Surgery, West China Hospital, Sichuan University, Chengdu, P.R. China (CF, CWF, YYY, CW, LY, ZGZ) and Institute of Digestive Surgery, State Key Laboratory of Biotherapy, West China Hospital, Sichuan University, Chengdu, P.R. China (CF, CWF, YL, ZGZ); and Laboratory of Stem Cell Biology, State Key Laboratory of Biotherapy, West China Hospital, Sichuan University, Chengdu, P.R. China (XMM).

## Abstract

Nonislet cell tumor hypoglycemia (NICTH) is a paraneoplastic syndrome characterized by persistent, severe hypoglycemia in different tumor types of mesochymal or epithelial origin; however, NICTH is infrequently induced by sarcomatoid carcinoma (SC). Despite some sarcomatoid and epithelioid characteristics in few cases of malignancies from epithelium, NICTH induced by recurrent SC in pelvic cavity in this report is extremely rare.

We report a case in which NICTH caused by recurrence and pulmonary metastases from SC in the pelvic cavity, and the computed tomography scan revealed multiple pelvic masses and multiple large masses in the pulmonary fields. During the treatment of intestinal obstruction, the patient presented paroxysmal loss of consciousness and sweating. Her glucose even reached 1.22 mmol/L while the serum glycosylated hemoglobin was normal and previous history of diabetes or use of oral hypoglycemic agents and insulin denied.

The laboratory examination showed that the low level of insulin, C-peptide, and growth hormone levels in the course of hypoglycemic episodes suggesting to the diagnosis of hypoglycemia induced by nonislet cell tumor, and the decreased levels of insulin-like growth factor (IGF)-I and IGFBP3 and the high expression of big IGF-II in the serum further confirmed the diagnosis of NICTH. Because of the widely pelvic recurrence and pulmonary metastases were unresected, the patient was discharged from the hospital after 2 weeks treatment with dexamethasone and glucose and unfortunately died 1 week later.

NICTH caused by SC in the pelvic cavity is extremely rare case in clinical. The aim of this report was to present the importance to examine big IGF-II expression in patient's serum in order to reach the diagnosis of NICTH in cases of intractable cancer-associated hypoglycemia.

## INTRODUCTION

Nonislet cell tumor hypoglycemia (NICTH) is a paraneoplastic syndrome characterized by recurrent fasting hypoglycemia, which is thought to be caused by the excessive secretion of incompletely processed precursors of insulin-like growth factor (IGF)-II (high molecular weight IGF-II or big IGF-II) by the tumor into the circulation.^1–3^ IGF-II-induced NICTH typically occurs in patients with large slow-growing neoplasm. In particular, it has been rarely reported in tumors of epithelial origin, while mesochymal tumors account for more than 50% of such cases.^4^

Sarcomatoid carcinoma (SC) is an uncommon malignant neoplasm of primitive epithelial cell origin that displays both malignant epithelial and mesochymal characteristics.^5^ Clinically, they are usually aggressive tumors with early occurrence of distant metastasis. To our knowledge, SCs rarely occur in the pelvic cavity, with only 1 case reported in literature.^6^ Here, we report an extremely rare case in which NICTH caused by recurrence and pulmonary metastases from SC in the pelvic cavity. We also confirmed that the big IGF-II was associated with the hypoglycemia.

## CASE REPORT

**FIGURE 1:**
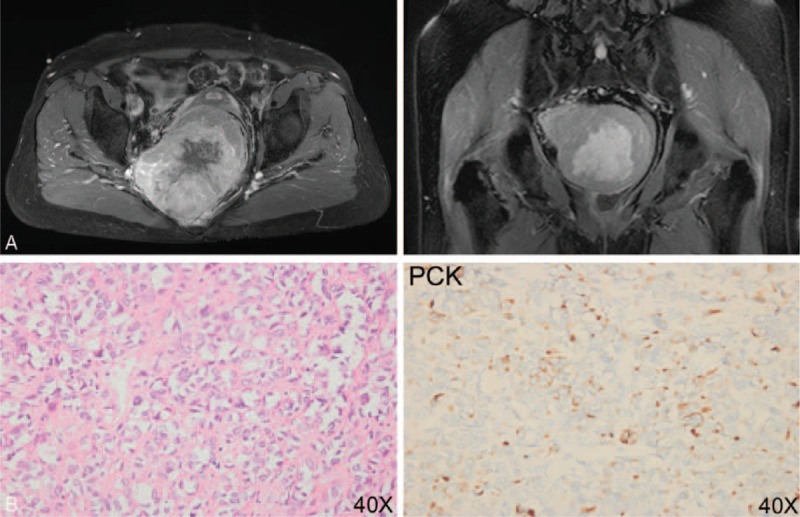
Diagnosis of PCSC. A: Abdominal MRI scan revealed a large (30 × 25 × 15 cm) heterogeneous pelvic mass and seemed to be widely adherent to rectum, posterior vaginal wall, and partial pelvic wall. B: Immunohistochemical results identified the diagnosis of sarcomatoid carcinoma/undifferentiated carcinoma (positive for PCK, weakly positive for EMA and CK7 and negative for CD125, WT1, D2-40, HMB-45, Caldesmon, Desmin, S100, DOG1, and CD117). MRI = magnetic resonance imaging; PCSC = SC in the pelvic cavity.

A 69-year-old woman received resection of pelvic mass at West China Hospital, Sichuan University in August 2013, following admission with difficult defecation and numbness of lower limb for 3 months. MRI scan at the time was showed in Figure 1A left and right. Postoperative histological examination revealed SC/undifferentiated carcinoma without invasion of rectum. The diagnosis was confirmed by immunohistochemical results (Figure 1B).

Postoperatively, the patient first received 2 cycles of TP regimen chemotherapy (cisplatin + paclitaxel); however, imaging assessment gave hints of disease progression with pelvic recurrence and pulmonary metastases. Thus, 2 cycles of second line MAID regimen (mesna + ifosfamidum + epirubicin hydrochloride for injection + dacarbazine) was given which also resulted in disease progression. At this point, the patient and family refused further chemotherapy and discharged.

**FIGURE 2:**
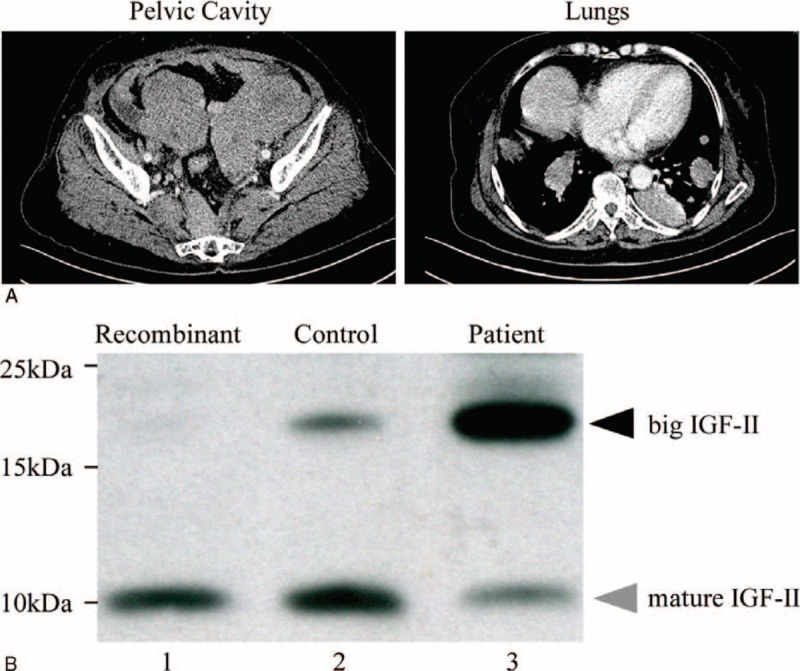
Diagnosis of NICTH. A: Abdominal CT scan revealed the pelvic recurrence and pulmonary metastases. B: Lane 3 showed that IGF-II was mainly detected as high molecular weight band in the sample from the patient, and lanes 2 and 3 showed the concentration of total IGF-II (ie, mature and big) of the patient's serum was higher than that in the control subject. CT = computed tomography; IGF-II = insulin-like growth factor II; NICTH = nonislet cell tumor hypoglycemia.

Twelve months later, the patient was admitted into the emergency because of intestinal obstruction. Computed tomography (CT) scan revealed multiple pelvic masses and multiple large masses in the pulmonary fields (Figure 2A left and right). This was treated by gastrointestinal decompression and parenteral nutrition. During this course, the patient presented paroxysmal loss of consciousness and sweating. Her glucose even reached 1.22 mmol/L while the serum glycosylated hemoglobin was normal (Table 1) and previous history of diabetes or use of oral hypoglycemic agents and insulin denied. Successive synchronous blood measurements during hypoglycemic episodes showed low insulin, C-peptide, growth hormone (GH), IGF-I, and IGF-binding protein-3 (IGFBP3) levels in favor of NICTH (Table 1).

**TABLE 1:**
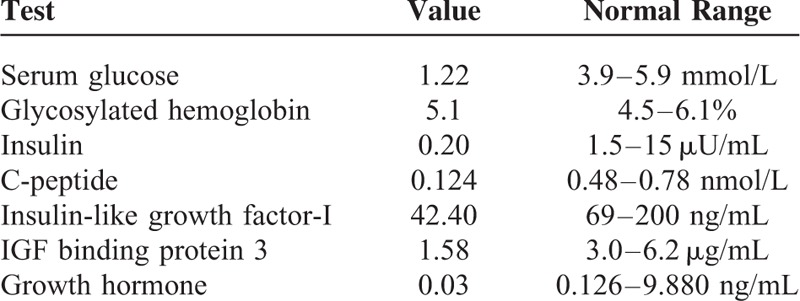
Laboratory Examination for the Patient

Later on, western blot of serum sample was conducted with an anti-IGF-II antibody which detected a high percentage of high molecular weight band IGF-II. Moreover, compared with control, the concentration of total IGF-II (ie, mature and big) was higher (Figure 2B, lanes 2 and 3).

Because of the widely pelvic recurrence and pulmonary metastases were unresected, treatment with oral dexamethasone (2 mg/day) and continuous intravenous infusion of glucose (150 g/day) were conducted. Dexamethasone and glucose doses were administrated to 1 mg/day and 200 g/day, respectively, to prevent recurrent hypoglycemia. The patient was discharged from the hospital and unfortunately died 1 week later.

## DISCUSSION

Hypoglycemia not associated with diabetes and/or its therapy is an uncommon clinical disorder, and tumor-induced hypoglycemia (TIH) caused by different mechanisms is a rare clinical entity.^7–9^ TIH usually appears as a result of insulin hypersecretion by pancreatic islet β-cell tumor (insulinoma), and can also be developed by other nonpancreatic tumors (NICTH).^9^ The serum glycemia, insulin, C-peptide levels could be used for the diagnosis of insulinoma, and the serum GH, IGF-I, IGFBP3, and IGF-II, especially big IGF-II levels could be used for the diagnosis of NICTH.^3,7–10^

NICTH is a paraneoplastic syndrome characterized by persistent, severe hypoglycemia with various tumor types of mesochymal or epithelial origin.^1,7^ It can result in an emergent and life-threatening condition, which should be suspected when a patient with various solid tumors suffered from hypoglycemia.^8^ Although few cases of malignancies with both sarcomatoid and epithelioid characteristics were reported, such as renal cell carcinoma^1^ and thyroid carcinoma,^4^ a case of NICTH with recurrent PCSC in the present report is extremely rare. Moreover, further laboratory examination showed normalized serum glycosylated hemoglobin and low insulin, C-peptide, GH, IGF-I, and IGFBP3 levels which were in accordance with NICTH.

Nevertheless, the crucial event in the development of NICTH has been linked to the aberrant secretion of big IGF-II, the alterations in the somatotroph axis and the levels of circulating IGF binding proteins (IGFBPs).^10,11^ Detection of a high percentage of IGF-II as big IGF-II would guarantee the diagnosis of NICTH.^8^ Here in this report, the expression of big IGF-II was significantly more than the mature isoform. Theoretically, both IGF-I and IGF-II are capable of decreasing glucose levels, but, they fail to do so because they are normally trapped within the vascular vessel in a high molecular weight protein complex with IGFBP3 and acid-labile subunit (ALS).^12–14^ The big IGF-II reside in the binary complex with IGFBP3 which was considered to be capable of crossing the endothelial barrier, resulting in a persistent insulin-like activity and leading to severe hypoglycemia.^15,16^

It is well known that NICTH often occurs in patient with large tumors and it might be necessary for big IGF-II to reach a certain level in causing hypoglycemia.^9,10^ In this case, hypoglycemia never occurred when the primary PCSC was >30 cm in diameter. Instead, the recurrence and pulmonary metastases with a diameter of <11 cm led to NICTH. Interestingly, such phenomenon was also observed in renal cell carcinoma^1^ and thyroid carcinoma,^4^ which was caused by the over-expressed big IGF-II in both recurrent tumor and the primary tumor.

In conclusion, this is an extremely rare case of SC in the pelvic cavity with the NICTH syndrome caused by inhibition of the somatotroph axis resulting from excessive secretion of big IGF-II. It is important to examine big IGF-II expression in patient's serum in order to reach the diagnosis of NICTH in cases of intractable cancer-associated hypoglycemia.
